# Clear Aligners in Pediatric Dentistry: A Scoping Review

**DOI:** 10.7759/cureus.58992

**Published:** 2024-04-25

**Authors:** Aakriti Chandra, Nilima R Thosar, Himani Parakh

**Affiliations:** 1 Pediatric and Preventive Dentistry, Sharad Pawar Dental College and Hospital, Datta Meghe Institute of Higher Education and Research, Wardha, IND

**Keywords:** esthetic correction, mixed dentition, orthodontic treatment, pediatric dentistry, clear aligner

## Abstract

Today not just adults but also children are affected by their looks and appearance. Their facial and dental appearance primarily influence how they present themselves in the outside world. Poor esthetic appearance at any age, especially when it comes to children, affects their psychological status. In earlier times, correction of dentition used to be done with crude methods, after which came the concept of braces, which were fixed on the labial surfaces. Even with these, the patients are equally concerned with their looks throughout the treatment, and thus neither adults nor children are eager to use the standard metallic-looking orthodontic gear. To tackle this problem, researchers have developed several solutions, and clear aligners are the modern and aesthetic answer. For effective tooth movement into the desired position, thin, transparent, plastic aligners known as invisible aligners are used, which are created using the computer-aided design/computer-aided manufacturing (CAD-CAM) technology. These aligners are analogous to the splints that cover the clinical crowns as well as the marginal gingiva. The treatment requires proper patient motivation as there can be poor compliance by patients. However, it can offer greater dental hygiene, comfort, and an excellent aesthetic experience during treatment.

This review highlights the history of fabrication of clear aligners, examining the efficacy, advantages, and disadvantages of transparent aligners for pediatric patients and also dentists. It weighs aspects like aesthetic appeal, comfort, oral hygiene, treatment predictability, and practice efficiency when comparing transparent aligners, like Invisalign, to conventional orthodontic treatments. It also ascertains the applicability and worth of clear aligners in contemporary orthodontic practice, while examining patient happiness, compliance, and overall treatment results.

## Introduction and background

After caries and periodontal diseases, malocclusion is considered the third most common dental problem worldwide [1]. When talking about young adults and children, it is the second most prevalent dental condition that affects around 30%-40% of children, compromising both appearance and dentofacial apparatus functionality [2]. Malocclusion has several negative effects, including periodontal diseases, problems with chewing, speaking, and swallowing, an increased risk of trauma, and poor aesthetics. It has been proven that people with esthetic smiles and proper occlusion have higher confidence and health-related quality of life than those with malocclusion [3]. Not only lives of adults, but also those of children are affected by this. When we talk about the methods of correction of malocclusion, the thought of braces and metallic wires comes to mind. Poor aesthetic appeal affects psychological well-being at any age, particularly in children. Before the invention of braces that were fixed to the labial surfaces, crude methods were used for correction. However, patients are equally concerned with their appearance during this treatment, so neither adults nor children are eager to wear the conventional metallic-looking orthodontic equipment. Hence, researchers have come up with several treatment solutions to address the growing aesthetic need when it comes to an alternative to traditional braces, including composite braces, and ceramic and lingual orthodontics, but all of them come with their own disadvantages. As a modern method for non-extraction cases, an aesthetic treatment solution was introduced known as clear aligners. These can be used for malaligned and mild crowded teeth, a deep overbite, spacing problems, narrow arches, etc. [4]. For effective tooth movement into the desired position, a thin, transparent, plastic aligner, known as Invisalign has been developed. To create an accurate cast that can be scanned to create a virtual 3D model, impressions are obtained. Using proprietary software, the dentist can edit this 3D model and almost completely cure malocclusion (about 89%) [5]. Then, it can be utilized to create a series of transparent plastic aligners that gradually correct the malocclusion. These aligners are made using the computer-aided design/computer-aided manufacturing (CAD-CAM) technology analogous to the splints that cover the clinical crowns as well as the marginal gingiva. Excellent observance is mandatory as the aligners have to be worn for a minimum of 20 to 22 hours a day and a total of about 400 hours to be effective [6]. Thus, the objective of this review was to assess elements like comfort, hygiene conditions, efficiency, predictability, and aesthetics when it comes to clear aligners. Furthermore, it evaluated patients' happiness and compliance, and treatment outcomes in order to determine the suitability and worth of clear aligners in modern orthodontic practice.

Methodology

For this review on the role of clear aligner therapy (CAT), a thorough literature search was carried out for years 1945 up to 2023, utilizing various electronic databases such as Scopus, PubMed, and Google Scholar using the following keywords: Clear aligner OR Aligners OR Invisalign OR CAT OR Pediatric Dentistry OR Orthodontics OR Ortho Correction OR Mild Crowding OR Mixed Dentition OR Primary Dentition OR Pedodontics. We made sure that the articles searched were published in the English language. The gathered information was then organized and narratively combined to highlight the key findings from moderate and high-quality reviews.

## Review

History

Clear aligners were first introduced in 1945 by Kesling [7]. Later in 1964, Nahuom created a dental contour appliance for orthodontic tooth movement using thermoformed plastic sheets [8]. However, in 1993, it was further modified and named Essix appliance by Sheridan et al. [9]. Furthermore, in 1997, two graduates Zia Chishti and Kelsey Wirth from Stanford founded Align Technology (San Jose, CA); they developed a digitally created clear aligner and called it Invisalign. Later in 2005, a new competitor entered the market, OrthoClear®, which was independently developed by one of the founders of Align, Zia Chishti. A clear aligner is a removable orthodontic appliance/gear that has become a popular treatment option in recent decades to correct dental overlaps and malalignment [6].

Materials used in clear aligners

Polyethylene terephthalate glycol (PET-G) remains the most commonly used material in clear aligners. Other materials include polypropylene, polycarbonate, thermoplastic polyurethanes, and ethylene vinyl acetate (Table 1) [10].

**Table 1:** Materials used by different brands of clear aligners Source: Ref. [6]

Product name	Components
Bioplast (Scheu-Dental, GmbH, Iserlohn, Germany)	Ethylene-vinyl acetate copolymer
Copyplast (Scheu-Dental, GmbH)	Polyethylene
Duran (Scheu-Dental, GmbH)	Polyethylene terephthalate glycol
Hardcast (Scheu-Dental, GmbH)	Polypropylene
Imprelon S (Scheu-Dental, GmbH)	Polycarbonate
Essix A+ (Dentsply Raintree Essix, Inc.)	Copolyester
Essix G+ (Dentsply Raintree Essix, Inc.)	Polypropylene/ethylene copolymer (>95%)
Invisalign (Align Technology, San Jose, CA)	Polyurethane from methylene diphenyl diisocyanate and 1,6-hexanediol

Digital patient impressions are captured by an intraoral scanner (iTero intraoral scanner; Align Technology) and uploaded on ClinCheck software (Align Technology) for online treatment planning. For traditional impressions made using polyvinyl siloxane putty, casts are acquired, scanned, and uploaded. Interproximal reduction (IPR) and expansion requirements for digital models are next examined for digital sectioning of teeth. After that, the teeth are digitally realigned, and the finished model is placed over the original model to produce a digital overlay model. A series of aligners is made utilizing the overlay model. Next, the force bumps, attachments, and auxiliary movements are planned to help the tooth movements [6].

Indications

In orthodontics, various dental malocclusions may necessitate intervention to achieve optimal alignment and functional harmony. Mild crowding, characterized by a spacing discrepancy of 1-5 mm between teeth, poses a common challenge that orthodontists address [4]. Similarly, spacing problems within the same range may also arise, requiring corrective measures to establish proper dental alignment. Deep overbite cases, particularly those falling under Class II, division 2, involve an excessive overlap of the upper front teeth over the lower counterparts. Narrow arches, indicative of insufficient space for proper dental alignment, warrant attention to enhance both aesthetics and functionality. Distal tipping of molars, a condition where the molars tilt towards the back of the mouth, can be addressed through orthodontic interventions. Additionally, anterior crossbites, where the upper front teeth sit behind the lower front teeth, necessitate corrective measures to achieve a harmonious bite. Orthodontic treatments tailored to these indications aim to optimize dental alignment, improve oral function, and enhance the overall oral health and aesthetics of individuals [11].

Indications for CAT in mixed dentition

Clear aligner therapy emerges as a viable option for addressing mild to moderate malocclusions in children, encompassing issues such as crowding, spacing, and minor corrections. Beyond their functional benefits, clear aligners offer a visually discreet alternative to traditional braces, making them particularly appealing to both children and parents concerned about the aesthetic impact of orthodontic treatment. This preference for clear aligners is rooted in their subtlety, contributing to a positive impact on a child's self-esteem throughout the treatment. Additionally, clear aligners can play a role in early intervention during the mixed dentition stage, providing a proactive approach to address orthodontic concerns before they exacerbate. However, the success of CAT in children hinges on their ability to adhere to the prescribed wear schedule, underscoring the importance of compliance for achieving optimal treatment outcomes [6].

The application of invisible aligners in pediatric patients has been explained in various studies with different cases as mentioned in Table 2.

**Table 2:** Application of CAT in pediatric patients CAT, clear aligner therapy

Authors and year	Study	Conclusion
Abraham et al., 2016 [12]	"Modified clear tray aligners" were utilized to rectify the anterior crossbite in an 8-year-old child.	The acceptance of treatment has increasingly emphasized aesthetics and convenience, as children have become highly conscious of their appearance when wearing orthodontic appliances.
Staderini et al., 2020 [13]	Two 8-year-old children with an anterior crossbite had successful treatment with CAT, with no major discomfort or consequences, after the problem was fully resolved in five months.	Given this result, CAT may be especially useful for correcting teeth that are still growing and flexible enough to realign.
Levrini et al., 2021 [14]	A total of 20 patients with a mean age of 8.9 years were treated with Invisalign® First for maxillary expansion and showed significant increases in arch width and perimeter, while arch depth and molar inclination decreased. The alveolar expansion was observed at all reference points.	The study suggests Invisalign® First clear aligners as a reasonable alternative for mild crowding or limited transverse maxillary deficiency compared to traditional expanders.
Zou et al., 2022 [15]	A 4-year-old child with an anterior crossbite and facial asymmetry was treated with CAT for about 18 weeks.	It showed great potential in early intervention for malocclusion in deciduous teeth.
Lombardo et al., 2023 [16]	A total of 32 children were given two different orthodontic expansion treatments (17 children: rapid maxillary expansion; 15 children: clear aligners) for the correction of malocclusions.	A clear aligner system was found to be more efficient than rapid maxillary expansion with better control of upper first molar crown angulation and increased palatal area; the anterior segment of the arch could achieve greater expansion.
Lione et al., 2023 [17]	A total of 23 subjects with a mean age of 9 years underwent non-extraction treatment with Invisalign First System® clear aligners to assess the transverse development of the maxillary arch.	For individuals who needed to develop their maxillary arches, the Invisalign First System® worked well. The largest net increase was seen in the upper first deciduous molars, and rotational movement around the palatal root resulted in an increased mesial breadth in the upper first molars.
da Silva et al., 2023 [18]	A total of 32 patients with a mean age of 9.3 years were treated with a 2x4 fixed appliance and clear aligners for about 8 months for the correction of maxillary incisor position irregularities in mixed dentition.	Clear aligners and 2×4 mechanics showed comparable efficacy and efficiency in correcting maxillary incisor positions during the mixed dentition phase.

Contraindications

Contraindications include open bite cases, cases requiring teeth extrusion, cases requiring more than one missing tooth, teeth with short clinical crowns, crowding or spacing issues of more than 5 mm, anteroposterior skeletal issues of more than 2 mm, centric relation, and centric occlusion discrepancies, severely rotated and tipped teeth, and more [19].

Practitioner perspectives and considerations

Practitioners undertaking the use of clear aligners in pediatric cases must consider several crucial aspects throughout the treatment process. Firstly, a comprehensive patient evaluation by an orthodontist or pediatric dentist is essential to determine the suitability of clear aligners for a child with mixed dentition. This assessment encompasses the child's dental development, orthodontic requirements, and existing oral hygiene practices. Subsequently, a personalized treatment plan is crafted, outlining the number of aligners needed, the expected duration of therapy, and the anticipated results. This strategy considers the child's ongoing growth and the eruption of new teeth. Parental involvement is imperative, with parents playing a crucial role in supporting their child's healing by ensuring good oral hygiene practices and consistent wear of aligners throughout the prescribed duration. Regular follow-up appointments are scheduled to monitor progress and make necessary adjustments, recognizing the dynamic nature of children's developing mouths. It is crucial to seek the expertise of a practitioner specializing in pediatric dentistry or one experienced in managing mixed dentition challenges. The practitioner should also provide comprehensive orthodontic education to both parents and children, explaining the treatment strategy, setting expectations, and underscoring the importance of follow-up. Additionally, practitioners must explore alternative options such as conventional braces if specific difficulties arise during the dentition phase. Finally, the retention phase post-treatment is emphasized, often involving the use of retainers to maintain the achieved orthodontic gains, depending on the individual circumstances [19].

Clear aligner therapy is an appropriate treatment when used correctly. However, achieving results and giving a child a healthy and harmonious smile requires preparation, adherence to treatment guidelines, and ongoing monitoring.

Advantages

Clear aligners present a range of advantages that contribute to their popularity as an orthodontic treatment option. One notable benefit is their aesthetic appearance, characterized by transparency and diminished visibility compared to traditional braces, making them a preferred choice for those seeking a discreet approach to orthodontic correction. In terms of comfort, clear aligners lack the metal brackets and wires that can cause discomfort and mouth lacerations associated with braces, rendering them generally comfortable to wear. The near invisibility of aligners allows patients to maintain confidence in their smiles throughout the treatment process. From a technical standpoint, orthodontists find it easier to work with clear aligners compared to lingual appliances or traditional braces. Clear aligners also offer improved oral hygiene as they can be removed for cleaning, preventing the trapping of food particles commonly associated with fixed braces. The option for retreatment using clear aligners facilitates modifications or addresses recurring orthodontic difficulties. Clear aligner appointments are typically simpler and shorter than those for traditional braces, providing a convenient advantage. Furthermore, clear aligners offer a more precise estimate of treatment duration compared to braces. They may also eliminate the need for premolar extractions to create interproximal space between teeth. Lastly, the patient's independence in changing aligners every few weeks contributes to a reduction in the frequency of dental visits, enhancing convenience in the orthodontic treatment process [20].

Disadvantages

Clear aligner therapy comes with certain disadvantages that individuals should consider before opting for this orthodontic treatment. Patients may suffer negative consequences if they do not wear their aligners as directed on a regular basis. One significant disadvantage is the necessity for rigorous removability [4,20]. Patient motivation plays a crucial role, as individuals need to be self-disciplined and committed to following the prescribed usage guidelines for the treatment to be effective. The time commitment associated with wearing clear aligners can be substantial, as they are typically recommended to be worn for approximately 22 hours every day. The inconvenience of removing aligners for meals is another factor to consider. Issues related to compliance, such as poor dental hygiene or missed appointments, oral hygiene requirements and the likelihood of dental discomfort may arise as a result of interproximal reduction resulting in less successful outcomes and prolonged treatment timeframes. Additionally, the cost of CAT may exceed that of traditional braces, varying depending on the location of the patient and specific characteristics of each case. It is essential for individuals to weigh these disadvantages against the potential benefits before deciding on the most suitable orthodontic option for their needs [20].

Fabrication of clear aligners

The creation of clear aligners involves both manual and digital processes. Clear aligners, which progressively move teeth into the proper positions through orthodontics, have become more and more popular because they look better than traditional braces [9,21,22]. Next, we delve deeper into the two fabrication techniques.

Manual Fabrication

Manual fabrication of orthodontic aligners involves a meticulous step-by-step process aimed at achieving precise tooth alignment. The procedure begins with creating a wax setup for each tooth, and strategically repositioning them to achieve the desired alignment. Subsequently, the patient's dental impressions are used to produce a functional cast. Within this cast, the teeth are carefully extracted and repositioned in accordance with the predetermined alignment objectives. The realigned dental models serve as the basis for crafting aligner sheets, employing techniques such as pressure molding or vacuum forming. This fabrication method allows for the customization of aligners tailored to unique tooth movements required for each patient, facilitating a gradual and targeted shift toward the correct tooth positions over the course of treatment [9,21].

Digital (CAD-CAM) Fabrication

Digital (CAD-CAM) fabrication revolutionized the creation of orthodontic aligners through a technologically advanced process. The initial phase involves generating precise digital impressions of the patient's teeth using an intraoral scanner, offering a comprehensive three-dimensional depiction of the oral features. In cases where traditional polyvinyl siloxane putty is employed for impressions, these are scanned and transformed into digital models. The digital models serve as a foundation for evaluating necessary tooth motions, including interproximal reduction and expansion requirements. The virtual execution of planned IPRs and digital tooth sectioning ensure authenticity and appropriateness. Subsequently, the teeth are digitally repositioned in alignment with the prescribed treatment plan. The creation of a digital overlay model involves superimposing the final digitally aligned model onto the original, serving as a guide for crafting a series of aligners, each representing a distinct stage in the treatment plan. To enhance tooth movement, additional components such as force bumps, attachments, or auxiliaries can be precisely fabricated through the digital process, contributing to the overall efficacy and customization of the orthodontic treatment [22].

Due to its accuracy and efficiency, digital manufacturing with CAD-CAM has grown in popularity in recent years, given the advantages of an easy, digitally guided presentation that can do a lot for accurate and predictable results in a clearly synchronized treatment plan. Manual fabrication methods, on the other hand, may still be used in certain situations but are generally considered less precise and more labor-intensive.

Wear time of clear aligners

A consistent and prolonged wear of clear aligners for 22 hours per day is essential to achieve the best results in orthodontic treatment [12]. The entire two-week period is usually advised to guarantee that all intended tooth motions are accomplished successfully and safely, even though some movements might happen more quickly during the first week. Treatment outcomes may be jeopardized if the recommended wear time is not followed [9]. To achieve the greatest possible benefit from clear aligner therapy, patients should diligently adhere to the advice provided by their orthodontist.

Principles of CAT

To properly straighten misaligned teeth, CAT uses two main principles. The initial principle involves creating space within the dentures. Various methods can be utilized to expand the entrance, i.e., to create space, such as IPR, which enables alignment correction through selective removal when teeth are overcrowded or by carefully eliminating a small portion of the enamel situated between the teeth.

The second important concept in CAT relates to the force and pressure to be used to stimulate teeth. Clear aligners are designed to gradually move a tooth into its proper position by applying gentle, consistent pressure to specific teeth. Additional devices such as attachments, dimples, digital power chains, elastics, etc. can be used to enhance this process and provide greater stability for precise changes. Significant changes are made to the tension and direction applied to each tooth depending on its size, shape, and type of movement needed, as well as the condition of the surrounding periodontal tissues [9].

Clear aligners are preferred over conventional braces due to their comfort and non-invasive nature [23]. These recommendations form the basis for an individualized treatment plan that considers each patient’s unique dental needs and goals, resulting in predictable and progressive tooth fit throughout the operation.

Comfort and patient willingness in clear aligner treatment

Adult and teenage patients are mostly motivated by aesthetic considerations when choosing clear aligner therapy as their orthodontic treatment option of choice. Research indicates that the nearly indiscernible appearance of CAT, which offers a low-key replacement for traditional fixed appliances, draws people in [24,25]. Miller et al. conducted a study that examined the first week of orthodontic therapy using fixed appliances against CAT and found various benefits related to CAT. CAT patients experienced far less pain and discomfort in the initial stage, most likely because there were no metal brackets or wires to irritate them [26]. Because the aligners may be taken out while eating and drinking, CAT also offers practical advantages by enabling a more regular diet and dental hygiene regimen. Additionally, especially in patients who are adolescents, CAT increases psychosocial well-being by lowering social anxiety and increasing self-esteem [27]. In orthodontic patients who value comfort and aesthetics, CAT is seen as a preferred option due to its real-time benefits in addition to its aesthetic advantages.

Repositioning of teeth using clear aligners

The use of clear polyurethane trays instead of traditional braces for orthodontic treatment has raised questions over the trays' efficacy in realigning teeth. Retrusion is the most precisely controlled movement of the teeth that can be achieved using clear aligners; in descending order of precision, these movements include rotation, fan-type expansion, and protrusion [28].

Clear aligners have demonstrated the capacity to deliver satisfactory outcomes in adjusting the bucco-lingual inclination of maxillary and mandibular incisors, particularly in cases of mild-to-moderate malocclusions [29]. CAT is particularly recommended for non-extraction cases involving mild-to-moderate malocclusions in patients who have completed their growth phase. In situations requiring tooth extractions, proper root angulations can be achieved through the strategic use of suitable attachments and a comprehensive understanding of the aligner system [30]. Notably, the most predictable and controlled movements with clear aligners include maxillary molar distalization (moving molars backward by 2.5 mm) and premolar extraction space closure (closing gaps resulting from premolar extractions, up to 7 mm) [31]. However, it is important to acknowledge that the thickness of the aligners can lead to challenges, such as the loss of occlusal contacts, which may affect the final alignment of the occlusal plane.

Alteration of the root structure during clear aligner treatment

Root resorption is a concern in orthodontic treatment, often associated with fixed braces due to the application of substantial orthodontic forces. Regarding clear aligners (CAT), there is, however, disagreement over how they affect root resorption [32]. When utilizing clear aligners, there has been evidence of a decreased incidence and severity of root resorption; the teeth most frequently affected are the incisors. Additionally, research has demonstrated that the rates of root resorption for both mild orthodontic pressures and transparent aligners are comparable. Another study has supported this theory by demonstrating that the use of transparent aligners during orthodontic treatment lowers the frequency and severity of root resorption [33]. This suggests that transparent aligners may be more beneficial in reducing the risk of root resorption than traditional fixed braces.

## Conclusions

The aesthetic appeal of clear aligners cannot be denied because they are transparent. It should not be forgotten that children are also impacted by facial appearance in their personal lives; however, patients should receive comprehensive education about the pros and cons of clear aligner therapy. Clear aligners have many advantages, including improved dental hygiene, comfort during use, ease of use, and better aesthetics during treatment as there is no compromise with the beauty of the smile. However, it is important to consider each individual’s preferences and expectations when determining whether CAT is the best option. To ensure that every patient gets the best possible outcome, dental professionals must provide appropriate advice to patients.
